# One-Dimensional Porous Silicon Nanowires with Large Surface Area for Fast Charge–Discharge Lithium-Ion Batteries

**DOI:** 10.3390/nano8050285

**Published:** 2018-04-27

**Authors:** Xu Chen, Qinsong Bi, Muhammad Sajjad, Xu Wang, Yang Ren, Xiaowei Zhou, Wen Xu, Zhu Liu

**Affiliations:** 1 Department of Physics and Astronomy, Yunnan University, Kunming 650091, Yunnan, China; chenxu@ynu.edu.cn (X.C.); ggmbqs1011@gmail.com (Q.B.); sajjadfisica@gmail.com (M.S.); reny@lzu.edu.cn (Y.R.); 2 Materials Science and Engineering Program, University of Houston, Houston, TX 77204, USA; xwang65@uh.edu; 3 Micro and Nano-materials and Technology Key Laboratory of Yunnan Province, Kunming 650091, Yunnan, China

**Keywords:** mesoporous structure, high surface areas, ultrafast, long-cycling life

## Abstract

In this study, one-dimensional porous silicon nanowire (1D–PSiNW) arrays were fabricated by one-step metal-assisted chemical etching (MACE) to etch phosphorus-doped silicon wafers. The as-prepared mesoporous 1D–PSiNW arrays here had especially high specific surface areas of 323.47 m^2^·g^−1^ and were applied as anodes to achieve fast charge–discharge performance for lithium ion batteries (LIBs). The 1D–PSiNWs anodes with feature size of ~7 nm exhibited reversible specific capacity of 2061.1 mAh·g^−1^ after 1000 cycles at a high current density of 1.5 A·g^−1^. Moreover, under the ultrafast charge–discharge current rate of 16.0 A·g^−1^, the 1D–PSiNWs anodes still maintained 586.7 mAh·g^−1^ capacity even after 5000 cycles. This nanoporous 1D–PSiNW with high surface area is a potential anode candidate for the ultrafast charge–discharge in LIBs with high specific capacity and superior cycling performance.

## 1. Introduction

Nowadays, the latest research works have paid more attention to the increasing demand of energy storage devices in various fields, such as supercapacitors, electric vehicles, portable electronics, and lithium-ion batteries (LIBs) [1,2,3,4,5]. The LIBs, which possess significantly higher energy density compared to sodium-ion batteries, lead-acid batteries, and aqueous nickel-based systems, have become the dominant power storage device [6,7,8,9,10]. Many novel technologies have been applied to improve the cyclability and electrochemical performance of the anode materials for LIBs by designing and utilizing various nanostructures. Silicon is a promising anode for LIBs due to its highest theoretical specific capacity (4200.0 mAh·g^−1^) [11,12,13], ten times larger than that of the commercial graphite anode (372.0 mAh·g^−1^), with low charge–discharge potential (~0.4 V, vs. Li/Li^+^) [14,15,16,17,18,19]. However, the tremendous volume change (>300%) of silicon anodes results in pulverization during fast charge lithiation and discharge delithiation processes, hindering their application as anode materials for LIBs [20,21,22].

Up to now, numerous strategies have been proposed to address the pulverization of the silicon anode by utilizing 0D to 2D nanostructures, such as hollow nanospheres, nanowalls [9], nanorods [23], nanosheets [24], nanotubes [16], porous silicon [25,26,27,28,29,30,31,32,33], and other silicon-based composites including Si–carbon nanofibers [34], Si–C [35,36], Si–graphene [37], Cu–Si core–shell [38], and conducting polymers [39]. The nanostructured silicon as anodes for LIBs reported so far has achieved the high specific capacity of 1390~3200 mAh·g^−1^ and high specific surface area [11,12,20,40,41]. For instance, Li et al. fabricated silicon nanowires with the high surface area of 219.4 m^2^·g^−1^, which show encouraging cycling performance as an anode with reversible capacity of 2111 mAh·g^−1^ at a relatively small current density of 0.8 A·g^−1^ after 50 cycles [42]. Cui et al. first reported that LIBs using silicon nanowires (SiNWs) with diameter of around 50 nm as anodes could achieve the theoretical charge capacity (~3200.0 mAh·g^−1^), but only maintain a discharge capacity around 75% of its original value over 10 cycles under even the small specific current of 0.2 A·g^−1^ due to pulverization [41]. Peng et al. reported that the purely electroless-etched SiNWs anodes of LIBs could achieve the large discharge capacity of 0.55 mAh·cm^−2^ over three cycles [43]. To address these issues, Bao’s group increased the fast charge–discharge current of 4.0 A·g^−1^ to achieve reversible capacity of ~626.5 mAh·g^−1^ after 200 cycles, by fabricating a nanoporous silicon particle-coated carbon layer with the feature size of ~50 nm and high surface area (303.2 m^2^·g^−1^) [40]. The porous nanoparticle structure with small feature size and high surface area improved cycling ability, but with capacity degrading at fast charge–discharge current density due to the coated carbon hindering fast diffusion of Li^+^. Hence, nanoporous silicon structures without coating and with smaller feature size could obtain high capacity with superior cycling performance at fast charge–discharge current density.

**Figure 1:**
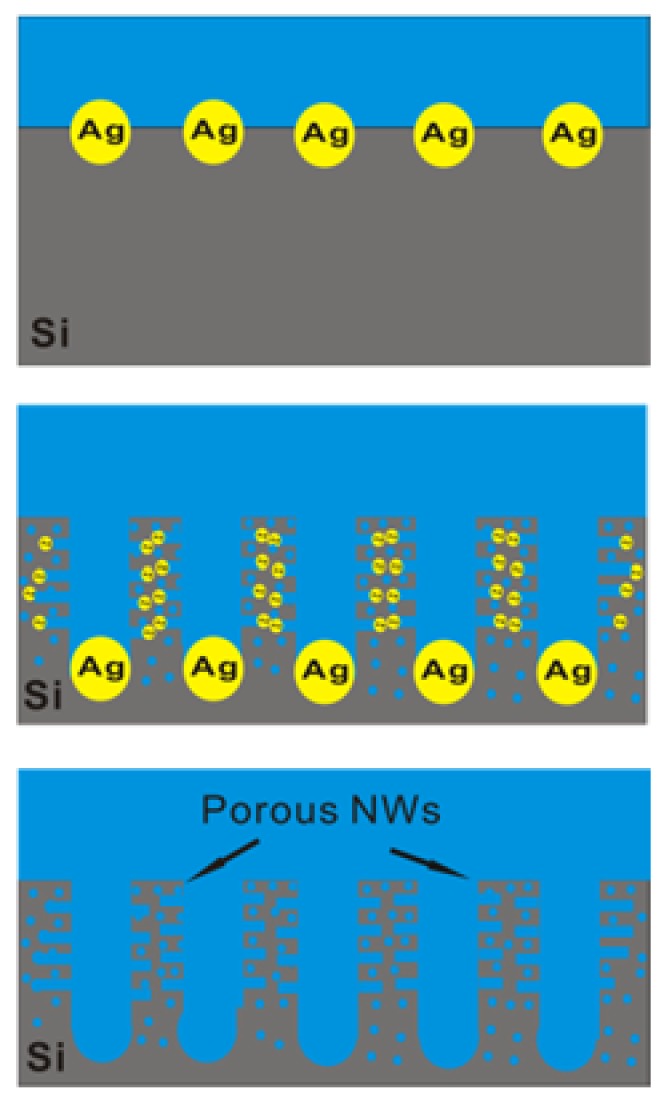
Schematic diagram illustrating the etching procedure of 1D–PSiNWs on silicon wafers.

Herein, we developed a novel 1D–PSiNWs anode with high specific area and small feature size without coating, to realize fast charge–discharge at the current density of 16.0 A·g^−1^. The schematic diagram of 1D–PSiNWs by one-step metal-assisted chemical etching (MACE) based on phosphorus-doped silicon wafers at 50 °C is shown in Figure 1 [44,45]. The formation mechanism of 1D–PSiNWs prepared by direct etching of phosphorus-doped silicon wafers is analyzed in Figure S1. It is noteworthy that the as-prepared 1D–PSiNWs have a high specific surface area of 323.47 m^2^·g^−1^ and a feature size of ~7 nm through the Brunauer–Emmett–Teller (BET) method. Moreover, those with optimized pore structure anodes exhibit reversible specific capacity of 2061.1 mAh·g^−1^ at a specific current of 1.5 A·g^−1^ after 1000 cycles. Our work provides a highly efficient way for the fabrication of fast charge–discharge anode materials for LIBs.

## 2. Experimental Methods

### 2.1. Preparation of 1D–PSiNWs

The preparation process of 1D–PsiNWs involves the use of one-step MACE of phosphorus-doped silicon wafers. N-type silicon wafers with <100> oriented (0.001–0.005 Ω · cm) were cut into pieces with measurements of 2.0 × 2.0 cm^2^ (Lijing Silicon Materials Co., Ltd., Quzhou, China). The fabrication process of 1D–PSiNWs was as follows: (1) the silicon wafer pieces were cleaned by ultrasonication in acetone (10 min), ethanol (10 min), and deionized water several times (DI water, 18.25 M Ω · cm), respectively. Then, silicon wafer pieces were dipped in H_2_SO_4_/H_2_O_2_ solution (volume ratio of 97% H_2_SO_4_/30% H_2_O_2_ = 3:1) at 96 °C (1 min) and completely washed with DI water. (2) Cleaned silicon wafer pieces were immersed in the mixture of 4.6 M HF solution and 0.02 M AgNO_3_ in sealed vessels and treated for 2 h at 50 °C in dark condition. Immediately, the thick dendritic silver film was coated on etched silicon substrates. (3) The etched silicon substrates were totally dipped into HNO_3_ (volume ratio of HNO_3_/DI water = 1:1) solution for 4 h to remove silver dendritic layer, then transferred into HF (5 wt %) solution to control 1D–PSiNW length and for a suitable time to ensure that the newly formed SiO_2_ was removed. The perpendicularly ordered 1D–PSiNWs could be discovered on silicon substrates after flaking off the dendritic silver film. (4) The resulting substrates were thoroughly washed with DI water and ethanol, and vacuum-dried at 70 °C for 8 h.

### 2.2. Material Characterization

The as-synthesized samples were characterized by an X-ray diffractometer (XRD, Rigaku TTRШ, Rigaku Corporation, Tokyo, Japan) using Cu-Kα radiation (1.5406 Å, 35 kV). The morphology and structure of the 1D–PSiNWs were investigated by scanning electron microscopy (SEM, FEI QUANTA200, FEI NanoPorts, Miami, FL, USA) coupled with energy dispersive X-ray spectroscopy (EDX), field emission scanning electron microscopy (FESEM, FEI Nova Nano SEM 450, Rigaku Corporation, Tokyo, Japan), and transmission electron microscopy (TEM, JEM-2100, Rigaku Corporation, Tokyo, Japan). Samples of TEM were obtained using ultrasonically oscillating etched silicon wafers in ethanol solution. The nitrogen adsorption and desorption isotherms were obtained using the Brunauer–Emmett–Teller (BET) method at 77 K after degassing the samples at 200 °C for 4 h by an analyzer (Quantachrome, Quadrasorb evo, Boynton Beach, FL, USA). The Barrett–Joyner–Halenda (BJH) method was applied to adsorption branches of isotherms to obtain pore diameter distributions. Chemical bonding analysis of the 1D–PSiNW surface layer was carried out using X-ray photoelectron spectroscopy (XPS, Thermo Scientific K-ALPHA^+^, Thermo Fisher Scientific, Waltham, MA, USA).

### 2.3. Electrochemical Characterization

The working electrodes were made up by mixing the active material (1D–PSiNWs), conductive agent (carbon black), and binder (sodium alginate) in a weight ratio of 50:30:20. The loading density of 1D–PSiNW active materials was ≈ 0.15 mg · cm^−2^. Coin-type cells (CR2025) were assembled in an argon-filled glove box (MIKROUNA super, O_2_ ≤ 0.1 ppm, H_2_O ≤ 0.1 ppm) while applying lithium metal as the counter electrode, a polypropylene (PP) microporous film as the separator, and LiPF_6_ (1 M) in ethylene carbonate (EC)-dimethyl carbonate (DMC) and diethyl carbonate (DEC) (volume ratio of EC:DMC:DEC = 1:1:1) as the electrolyte. The galvanostatic measurements were carried out on a LAND-CT2001A battery tester with a voltage window of 0.01–2.0 V at various current rates. The cyclic voltammetry was performed using an electrochemical workstation (chi604e) between 0.01 and 2.0 V at a scan rate of 0.1 mV · s^−1^. The electrochemical impedance spectra (EIS) of the cells were measured on the electrochemical workstation (chi604e) at the frequency range of 0.1 Hz–100 kHz. Nyquist plots derived from EIS were simulated by using Z-view software.

## 3. Result and Discussion

**Figure 2:**
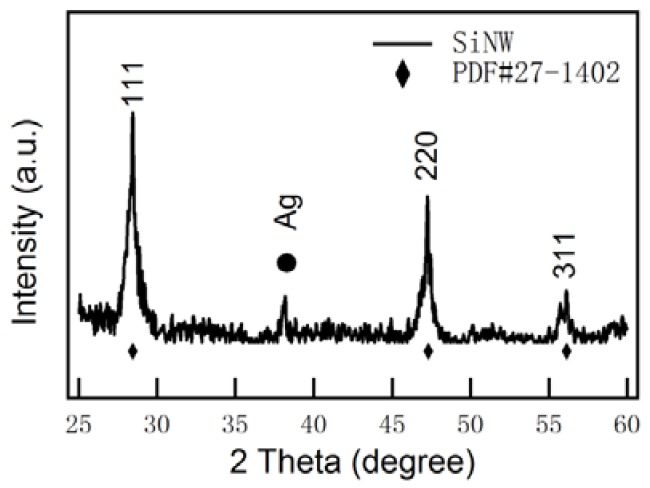
X-ray diffraction patterns of 1D–PSiNWs.

Figure 2 shows the XRD patterns of the 1D–PSiNWs. The main observed peaks shown in Figure 2 can be indexed to the face-centered-cubic (fcc) structure of silicon (JCPD Card No. 27-1402), which belongs to the space group Fd-3m (No. 227). The peak of 38.17° indicates the existence of a tiny minority of Ag in the obtained 1D–PSiNWs after HNO_3_ treatment. The morphologies and EDX analysis of 1D–PSiNWs coated by dendritic Ag film after etching were obtained by scanning electron microscopy (SEM) as presented in Figure S2a.

**Figure 3:**
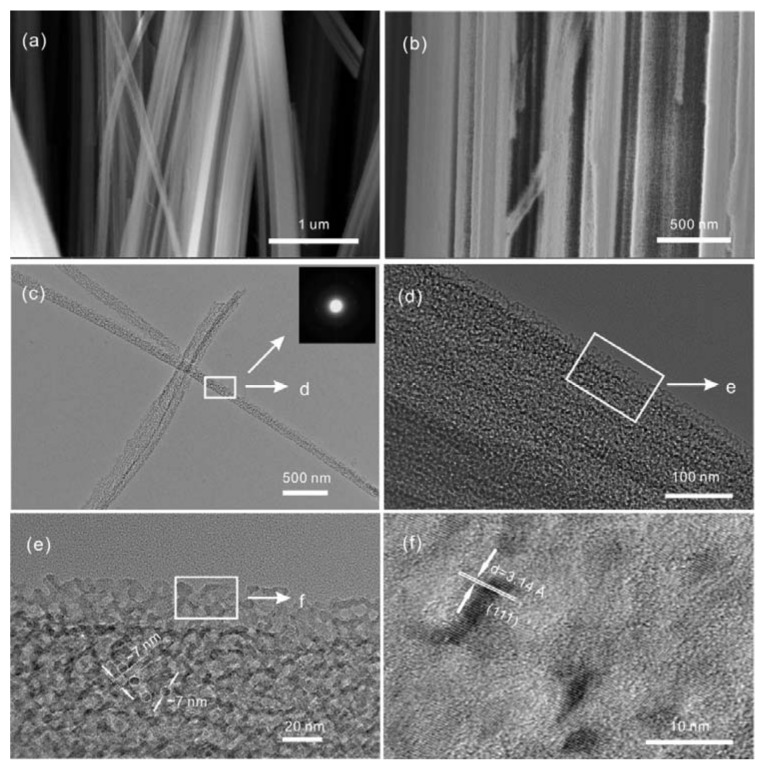
SEM (**a**,**b**), TEM (**c**,**d**), and HRTEM (**e**,**f**) images of 1D–PSiNW arrays etched.

High-quality 1D–PSiNW arrays can be produced on the n-type Si wafer by simply immersing the wafer into HF/AgNO_3_ solution for an appropriate etching time and temperature. Figure 3 shows FESEM (Figure 3a,b) and TEM (Figure 3c,f) images of 1D–PSiNWs. The cross-section profile in Figure 3a confirms that the 1D–PSiNW arrays are uniform on the entire wafer surface. The enlarge cross-section in Figure 3b indicates that the diameters of the vertically well-aligned arrays are in the range of 60.0–500.0 nm. Mesoporous holes are uniformly distributed on the surface of each nanowire. From the magnified cross-section image shown in Figure S2b, some orderly congregated bundles composed of 1D–PSiNWs can be clearly seen. The lengths of the as-prepared 1D–PSiNWs still near 7.0 μm after HF treatment. Figure S3c is an enlarged top view of 1D–PSiNWs, which indicates a uniform surface. The TEM images in Figure 3c–f demonstrate that the nanowires are highly porous at the surface, with both pore diameter and wall thickness around 7 nm. The polycrystalline structure was confirmed by the circular diffraction pattern in the selected area electron diffraction (SAED) taken on a single porous nanowire as illustrated in Figure 3c. Some parts of 1D–PSiNWs also have monocrystal properties, displaying clear lattice fringes corresponding to a silicon (111) lattice with an interplanar crystal spacing of 3.14 Å, as presented in Figure 3f. The phosphorus dopants provide electrons which facilitate the etching process and leave holes on the silicon nanowire surface, resulting in the coexistence of crystalline and amorphous states. Figure S1 illustrates a simplified quantitative model for the detailed growth mechanism of 1D–PSiNWs in aqueous HF/AgNO_3_ solution. The Fermi level of N-type silicon is more positive than the redox potential of Ag/Ag^+^, leading to majority carrier electrons which will transfer to the silicon/solution interface. Thus, the holes from the oxidant will be injected into the valence band of silicon with the Ag deposition or reduction of H^+^ which induces silicon substrate oxidization and dissolution, leading to SiNW growth. The etching process can be described as two main simultaneous electrochemical reactions [46,47]:

$${4\mathrm{Ag}}^{+}{+\;4e}^{-}\to 4\mathrm{Ag}$$

$${\mathrm{Si}+6F}^{-}\to {{[\mathrm{SiF}}_{6}]}^{2-}{+\;4e}^{-}$$

**Figure 4:**
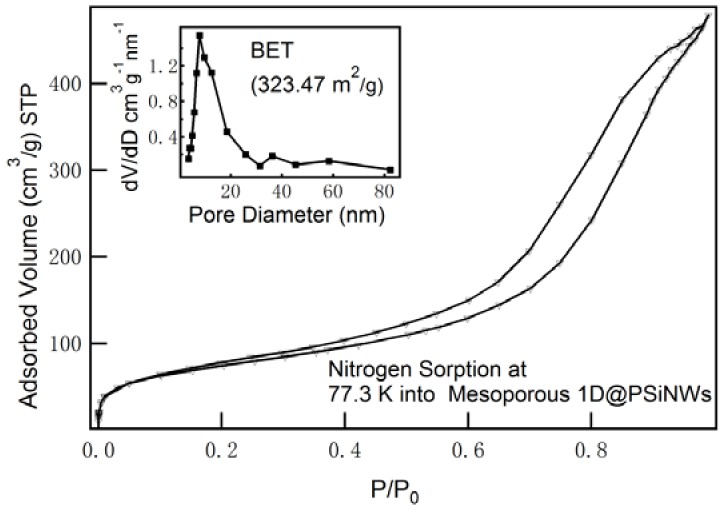
Nitrogen adsorption isotherm at 77.3 K (line, adsorption; triangular, desorption) and pore size distribution curve (inset) of 1D–PSiNWs depicted after fitting by the Barrett–Joyner–Halenda (BJH) model.

Surface area measurements are typically based on N_2_ sorption at 77.3 K, and the Brunauer–Emmer–Teller (BET) model is typically used to interpret the data. The N_2_ sorption isotherm and the pore diameter size distribution of 1D–PSiNWs are shown in Figure 4. Capillary condensation occurs in the higher P/P_0_ region (P/P_0_ > 0.15), and hysteresis can be observed, which conforms to the typical isothermal curve of mesopores (type IV). A pore size distribution analysis by Barrett–Joyner–Halenda (BJH) methods showed that there is a pore diameter distribution of 1D–PSiNWs which is mesoporous at the range of 3.8–80.5 nm. The maximum probability for feature size distribution is ~7 nm in diameter (inset). This result is in a good agreement with the TEM observation. The 1D–PSiNWs exhibit exceptionally high specific surface area of 323.47 m^2^·g^−1^. This value is, significantly, the same as the maximum value reported for pure porous silicon materials by metal-assisted chemical etching (MACE) [33].

**Figure 5:**
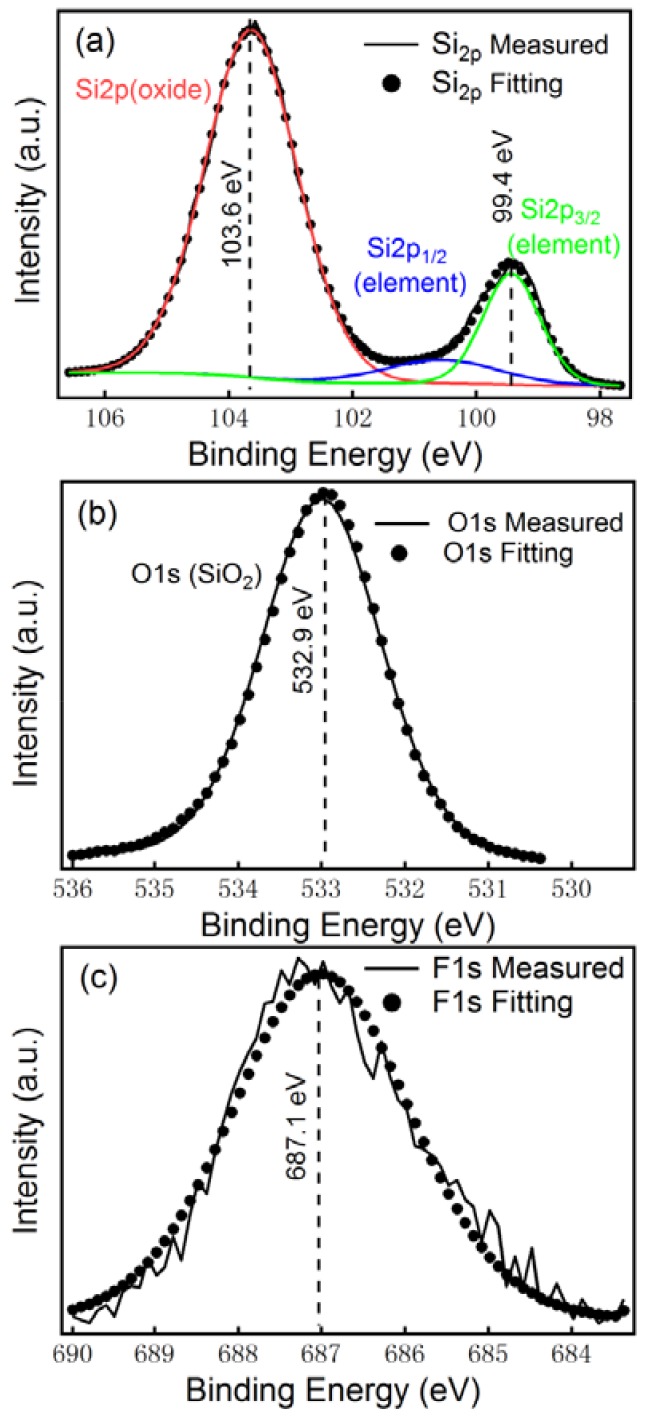
X-ray photoelectron spectroscopy (XPS) survey of 1D–PSiNWs using (**a**) Si_2p_, (**b**) O_1s_, and (**c**) F_1s_.

The whole XPS spectra of 1D–PSiNWs, which are calibrated with the C1s line of adventitious carbon at 284.8 eV, are presented in Figure S3 with the four main components fitted (Si at 99.4, SiO_2_ at 103.6, O_2_ at 532.9, and [SiF_6_]^2−^ at 687.1 eV) [22]. Figure 5a presents the Si_2p_-amplified high-resolution XPS spectrum, which reveals two peaks centered at 99.4 eV and 103.6 eV that can be attributed to silicon and SiO_2_ components, respectively. The typical Si_2p1/2_ and Si_2p3/2_ spectra are overlapped partly, giving an asymmetric peak shape at 99.4 eV. Figure 5b shows the O1s high-resolution XPS spectrum with a narrow signal peak at 532.9 eV, indicating the coexistence of unique oxygen chemical environments on the surface of the structure. The binding energy of the O1s signal at 532.9 eV is in full compliance with the chemical state of SiO_2_, which can be assigned to the Si–O bond. A weak additional peak corresponding to [SiF_6_]^2−^ at 687.1 eV is observed in Figure 5c. Actually, small quantities of organic fluorine which are not totally eliminated during the etching process are often found. In fact, as observed in the XPS spectra, the presence of oxygen atoms and fluorine atoms is due to the partial oxidation of 1D–PSiNWs surface and the formation of Si–O bonds/[SiF_6_]^2−^ during the etching process of the silicon wafer on the 1D–PSiNW surface. The XPS survey scans did not detect any other impurities except oxygen and fluorine. This also shows that the silver elements reflected by the XRD pattern do not exist on the surface of the 1D–PSiNWs, but inside the deep holes.

The electrochemical performances of 1D–PSiNWs were investigated by repeated charge–discharge cycling at room temperature in a coin-type half-cell using Li metal as the anode. The electrochemical reactions occurring in a coin-type half-cell is described by the following formula [32,48]:

$${\mathrm{Si}\;\mathrm{electrode}:\;\mathrm{xLi}}^{+}{+\;\mathrm{Si}+\mathrm{xe}}^{-}↔{\mathrm{Li}}_{x}\mathrm{Si},\;0\leq x\leq 4.4$$

$$\mathrm{Li}\;\mathrm{electrode}:\;\mathrm{Li}↔{\mathrm{Li}}^{+}{+\;e}^{-}$$

**Figure 6:**
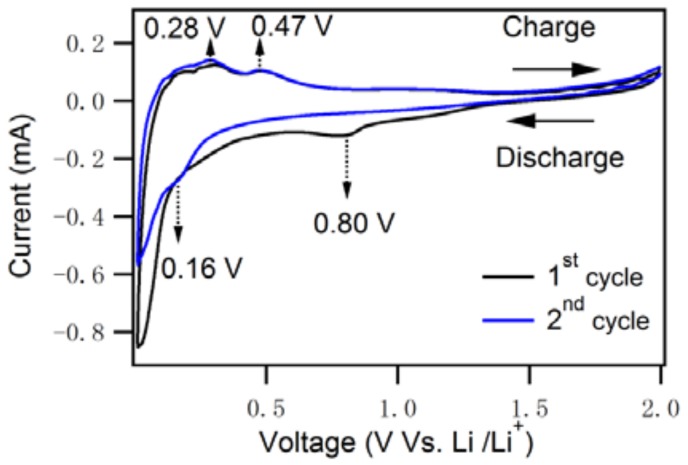
Cyclic voltammograms of 1D–PSiNWs anodes for the first and second cycle at scan rate of 0.1 mV·s^−1^ (voltage range: 0.01 V–2.0 V).

Cyclic voltammetry (CV) curves at the first and second charge–discharge cycles of anodes based on 1D–PSiNWs were acquired in the potential window from 0.01 V to 2.0 V (vs Li/Li^+^) at a scan rate of 0.1 mV·s^−1^ (Figure 6). The voltage profile observed was consistent with previous silicon anode studies, with a long flat plateau during the first charge, during which crystalline silicon reacted with Li^+^ to form amorphous Li_x_Si [12,40,41]. During the first discharge cycle, the peak at 0.8 V, which is absent at the second cycle, leads to the formation of an extremely large solid/electrolyte interphase (SEI) on the surface of the porous silicon nanowire electrode, which leads to irreversible capacity loss. We can see the peak at 0.16 V during the first discharge (Li alloy), which is due to the phase transition of silicon to the amorphous lithium-rich Li_15_Si_4_ structure. During the first charge process (Li dealloy), two broad peaks were observed at 0.28 V and 0.47 V, which can be attributed to the phase transition between amorphous Li_x_Si and amorphous silicon. Upon the second discharge and charge, peaks were also observed at 0.18 V and 0.28 V/0.47 V, which are the same as that of the first discharge, respectively. The CVs from the 200th to 203rd cycles are shown in Figure S4. The charge–discharge curves from the 200th to 203rd cycles almost overlap each other, thus indicating highly stable electrochemical cycling performance.

**Figure 7:**
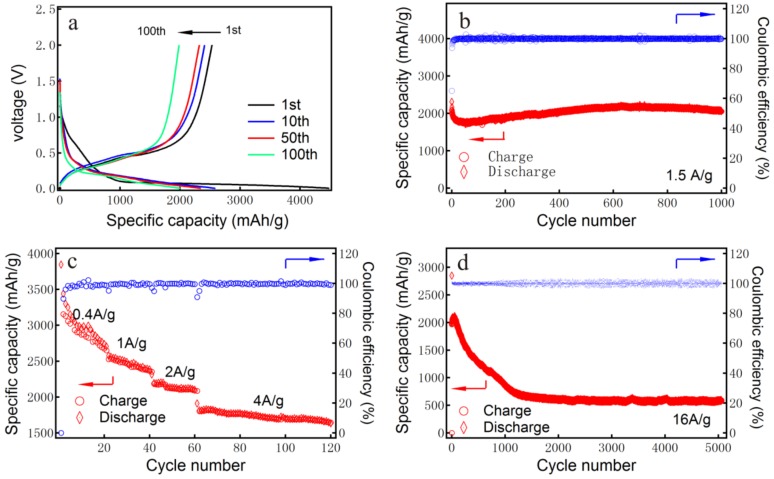
Results of electrochemical performance for 1D–PSiNW anodes. (**a**) Galvanostatic charge/discharge profiles between 0.01 V and 2.0 V vs. Li/Li^+^ for the first, 10th, 50th, and 100th cycles at a current density of 1.0 A·g^−1^. (**b**) Cycling performance of the as-prepared 1D–PSiNW anodes at a current density of 1.5 A·g^−1^. (**c**) Rate performance of 1D–PSiNW anodes at the current density of 0.4/1.0/2.0/4.0 A·g^−1^. (**d**) Electrochemical performance of 1D–PSiNW anodes with 5000 cycles at a current density of 16.0 A·g^−1^.

Representative charge–discharge profiles of the electrode based on 1D–PSiNWs with 100 cycles at a rate of 1 A·g^−1^ in the potential range of 0.01–2.0 V are shown in Figure 7a. The electrode based on 1D–PSiNWs exhibits a discharge capacity of 4487.2, 2592.0, 2350.3, and 2004.5 mAh·g^−1^ at the first, 10th, 50th, and 100th cycle, respectively. The first discharge and charge capacities of the as-prepared 1D–PSiNWs anodes are 4487.2 mAh·g^−1^ and 2534.5 mAh·g^−1^, respectively; thus indicating an initial coulombic efficiency of 56.5%. The irreversible capacity is due to the solid/electrolyte interphase (SEI) film forming on all surfaces of the 1D–PsiNWs with high specific surface area of 323.47 m^2^·g^−1^. Therefore, the first discharge irreversible capacity loss of ~1953.0 mAh·g^−1^ could mainly originate from the reduction of electrolyte and the formation of a SEI on the surface of an electrode, and from the irreversible insertion of lithium ions into silicon nanowires [42].

To show the advantage and stability of 1D–PSiNWs, the cycling performance is shown in Figure 7b. The 1D–PSiNW electrode exhibited excellent cycling performance with a reversible capacity of 2061.1 mAh·g^−1^ over 1000 cycles under a high current density of 1.5 A·g^−1^, with 99.7% coulombic efficiency. The rate capability (0.4–4.0 A·g^−1^) of the 1D–PSiNW anodes was evaluated in the voltage range of 0.01–2.0 V, as presented in Figure 7c. The specific discharge capacities of the 1D–PSiNWs for the last cycles are 2706.5, 2314.0, 2101.2, and 1667.0 mAh·g^−1^ at current densities of 0.4, 1.0, 2.0, and 4.0 A·g^−1^, respectively. After 60 charge–discharge cycles under a high current density of 4.0 A·g^−1^, a specific discharge capacity as high as 1667.0 mAh·g^−1^ was maintained. Increasing the current density to 8.0 A·g^−1^, a discharge capacity of 1650.6 mAh·g^−1^ after 60 cycles was delivered (Figure S5). After going through super-long 5000 cycles at 16.0 A·g^−1^, as shown in Figure 7d, a discharge capacity of 586.7 mAh·g^−1^ was still retained, which was much larger compared with that of graphite (~372.0 mAh·g^−1^). The slight increase in the capacity for 1D–PSiNWs in the initial 100 cycles is associated with the gradual activation of the silicon host (the same phenomenon can also be seen in Figure 7b). It is reported that there is a slight increase in the capacity until full lithiation of the active silicon [46,48,49,50]. The rapid fading in capacity for the silicon powder or nanowire was due to the large volume change during lithium alloying and dealloying processes, leading to fragmentation and an electrical disconnection between particles. The improved performance of pure 1D–PSiNWs can be attributed to the interconnected porous network structure, which offers sufficient void space to accommodate the large volume change. In addition, a huge quantity of mesopores inside silicon nanowires act as smooth channels for the fast Li^+^/electron transfer, and are conducive to the rate capability and cycling stability of 1D–PSiNW anodes.

**Figure 8:**
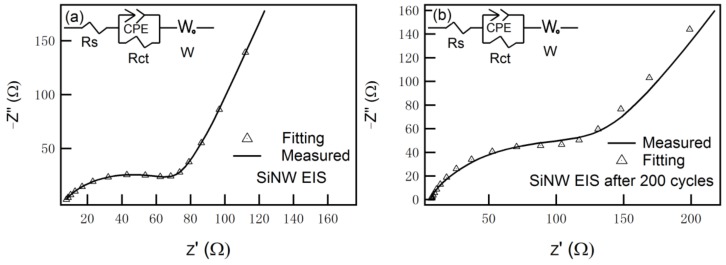
Typical electrochemical impedance spectra of 1D–PSiNW anodes measured at open-circuit voltage E ≈ 2.0 V (Li/Li^+^): (**a**) fresh cell; (**b**) after 200 cycles at a current density of 8.0 A·g^−1^ (inset is the equivalent circuit used to fit the electrochemical impedance (EIS)).

The electrochemical impedance (EIS) of the 1D–PSiNW electrode is investigated in Figure 8 to gain further insights into the improved cycling performance. The Nyquist plots obtained from open-circuit voltage (E = 2.0 V) manifested that the charge transfer resistance of the electrode is minimal for 1D–PSiNW anodes. The fitting equivalent circuit for the cell system is depicted in the inset of Figure 8. In the equivalent circuit for the fresh cell before cycling, shown in Figure 8a, R_s_ (7.7 Ω) is the bulk resistance of the 1D–PSiNW electrode, electrolyte, and separator, corresponding to the intercept value of the semicircle with the real axis at the high-frequency region. CPE is the constant phase element, and R_ct_ (66 Ω) is the charge transfer resistance, corresponding to the diameter of the semicircle at high frequency. W is the Warburg impedance arising from the semi-infinite diffusion of Li^+^ ions in the bulk of the electrode, which is generally indicated by a sloping straight line at the low-frequency region [51,52]. As shown in Figure 8b, the bulk resistance (R_s_) and the charge transfer resistance (R_ct_) are 6.7 Ω and 104 Ω for the 1D–PSiNW electrode after 200 cycles at a current density of 8.0 A·g^−1^, respectively. There is no significant resistance change in electrode conductivity after 200 cycles, indicating that the 1D–PSiNWs possess excellent conductivity retention. The 1D–PSiNW electrodes have excellent electron/ion conductivity.

## 4. Conclusions

In conclusion, we fabricated novel 1D–PSiNW anodes with the high specific area of 323.47 m^2^·g^−1^ by a one-step silver-assisted chemical etching method. The TEM image demonstrated that the nanowires were several µm length and 60–500 nm in diameter with highly uniform porosity at the surface, with both pore diameter and wall thickness around 7 nm. This small pore size of the silicon nanoporous structure offered sufficient void space to accommodate the large volume change. Our designed 1D–PSiNWs as anodes for LIBs show a reversible specific capacity of 2061.1 mAh·g^−1^ after 1000 cycles under a fast charge–discharge condition at 1.5 A·g^−1^. Even after 5000 cycles, a reversible capacity of 586.7 mAh·g^−1^ was retained at the ultrafast charge–discharge current density of 16.0 A·g^−1^. The small feature size acted as a short Li^+^/electron conductive path during the lithiation/delithation processes. The superior electrochemical performance and excellent cycling life of the nanoporous 1D–PSiNW anodes were attributed to the 1D structure with uniform interconnected nanoporous channels existing inside the silicon nanowires.

## Supplementary Materials

The following are available online at http://www.mdpi.com/2079-4991/8/5/285/s1. Figure S1: Energy-band diagrams of N-type silicon in aqueous HF/AgNO_3_ solution. Figure S2: (a) SEM and EDX of dendritic Ag-coated 1D–PSiNWs after etching, (b) SEM cross-section of 1D–PSiNWs, and (c) top view SEM image of 1D–PSiNWs. Figure S3: XPS survey spectra of 1D–PSiNWs. Figure S4: Cyclic voltammetry curves of 1D–PSiNW anodes of 200th, 201st, 202nd cycles in the voltage window from 0.01 V to 2.0 V at rate of 0.1 mV·s^−1^. Figure S5: The results of cycling performance of 1D–PSiNW anodes tested at a current density of 8.0 A·g^−1^.
