# Supplementary material for: One-Dimensional Porous Silicon Nanowires with Large Surface Area for Fast Charge–Discharge Lithium-Ion Batteries
Source: Nanomaterials (Basel). 2018 Apr 27;8(5):285. doi: 10.3390/nano8050285 (PMC5977299; doi:10.3390/nano8050285)
Supplement: Supplementary file 1 [file nanomaterials-08-00285-s001.pdf]

Supporting information

Figure S1 shows the energy band diagram of N-type silicon before contact with the HF/$AgNO_3$ solution (in the dark environment) and after contact with the Ag particles in the dark environment at room temperature. Electroless deposition of Ag on a silicon wafer surface in the HF/$AgNO_3$ solution is based on the galvanic replacement reaction, in which two simultaneous processes occur at the silicon surface: the cathodic reduction of Ag^+ ions (Equation (1)), which produces metallic Ag deposits; and the anodic oxidation of silicon as an electron-releasing reaction (Equation (2)), during which the charges released by the oxidation of silicon atoms are transferred to the sites of Ag deposition (SHE = standard hydrogen electrode, shown in Figure S1):

$$Ag^{+}+e^{-}\rightarrow Ag \quad E^{0}= +0.79 \text{ V/SHE} \quad (1)$$

$$Si+2F^{-}\rightarrow SiF_{2} +2e^{-} \quad E^{0}= -1.24 \text{ V/SHE} \quad (2)$$

The driving force of the charge exchange is the potential difference between the redox species and the Fermi level of silicon in the solution. The redox potential at equilibrium of each electrochemical reaction (Equation (1) / (2)) could be calculated using the Nernst equation (Equations (3) / (4)) as follows:

$$E_{Ag}(V/SHE)=0.80+\frac{RT}{F}Ln[Ag^{+}] \quad (3)$$

$$E_{Si}(V/SHE)=-1.24-\frac{RT}{F}Ln[F^{-}] \quad (4)$$

where E is the equilibrium potential, R is the ideal gas constant 8.314 $J\cdot K^{-1}\cdot mol^{-1}$, F is the Faraday constant 96485 $C\cdot mol^{-1}$, T is the absolute temperature, and [Ag^+] is the molarity of Ag^+ ions in the HF solution, respectively. The dissociation constant of hydrofluoric acid is assumed to be $K_1 = 6.8\times10^{-4}$ at room temperature. Thus, the concentration of hydrogen ions was calculated to be around 5.558×10^{-2} M. We can then obtain the equilibrium potentials E_{si} = 1.08 V (-5.58 eV). In the HF solution, the Ag^+ concentration is 0.02 M and the equilibrium potential of E_{Ag} is 0.69 V (-5.19 eV). The Fermi level of N-type silicon is E_{fN}= -4.13 eV, which is calculated from the donor atom carrier concentration (1.8×10^{18} atoms/cm^3), and the redox potential of the Ag/Ag^+ (-5.19 eV) in solution tends to align with the Fermi level of N-type silicon, as the silicon electrode was immersed into the solution. The Fermi level of N-type

silicon is more positive than the redox potential of Ag/Ag^+, leading to majority carrier electrons which will transfer to the silicon/solution interface. Thus, the holes from the oxidant will be injected into the valence band of silicon with the Ag deposition or reduction of H^+ which induces silicon substrate oxidization and dissolution, leading to SiNW growth. The Ag cannot be adsorbed randomly on the surface; the surface would be etched directionally instead of in the random fashion that leads to nanowire formation. The reduction of Ag happens very fast on both the silicon substrate and formed SiNW; numerous Ag nanoparticles are formed on the SiNW surface within seconds. The anodic oxidation of the SiNW surface dissolution should occur in the close vicinity of Ag nuclei to realize direct electron transport, which forms porous 1D nanowire structures.

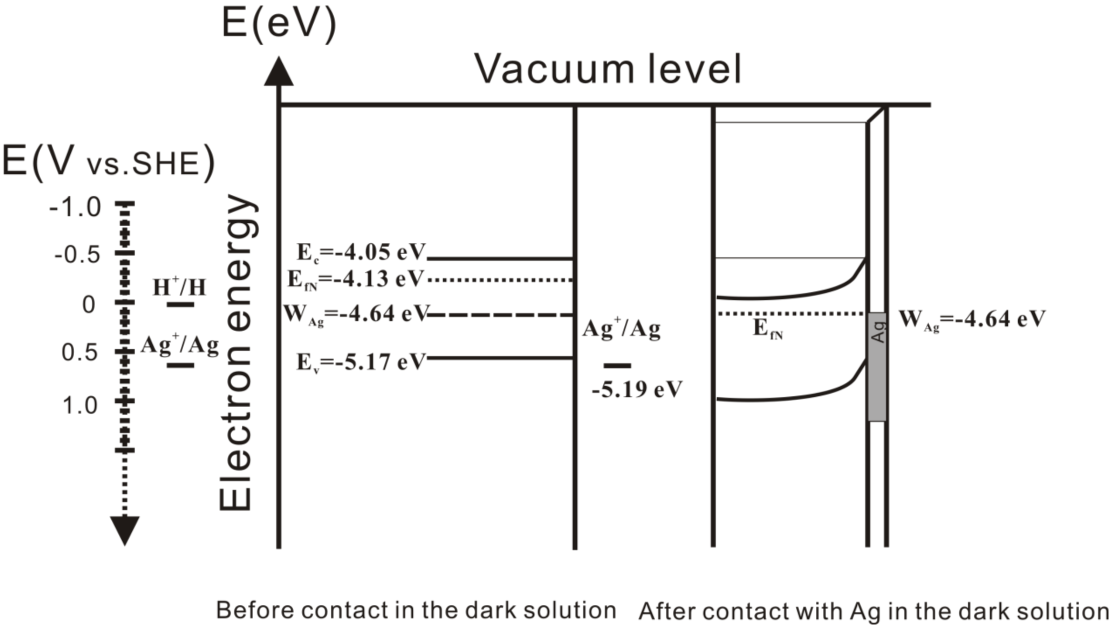

Figure S1. Energy-band diagrams of N-type silicon in aqueous $HF/AgNO_3$ solution. Typical values for energy levels are shown referenced to vacuum and to SHE. Left: before contact in the dark solution; right: after contact with Ag in the dark; energy-band bending of silicon and the formation of the quasi-Schottky Ag/Si interface.

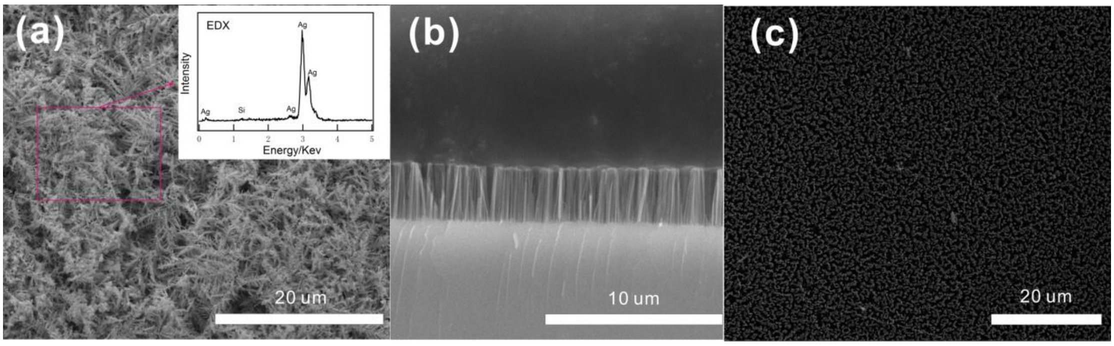

Figure S2. (a) SEM and EDX of dendritic Ag-coated 1D@PSiNWs after etching, (b) SEM cross-section of 1D@PSiNWs, and (c) top view SEM image of 1D@PSiNWs.

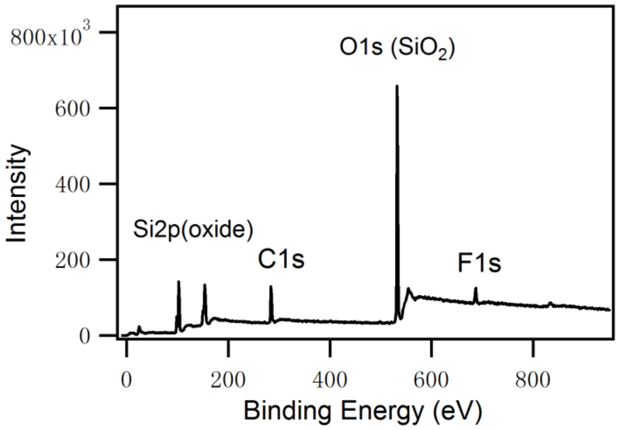

Figure S3. XPS survey spectra of 1D@PSiNWs.

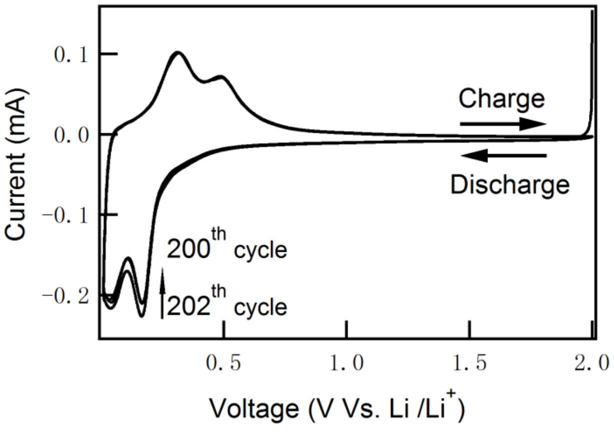

Figure S4. Cyclic voltammetry curves of 1D@PSiNW anodes of the 200th, 201st, and 202nd cycles in the voltage window from 0.01V to 2.0 V at the rate of 0.1 mV·s^{-1}.

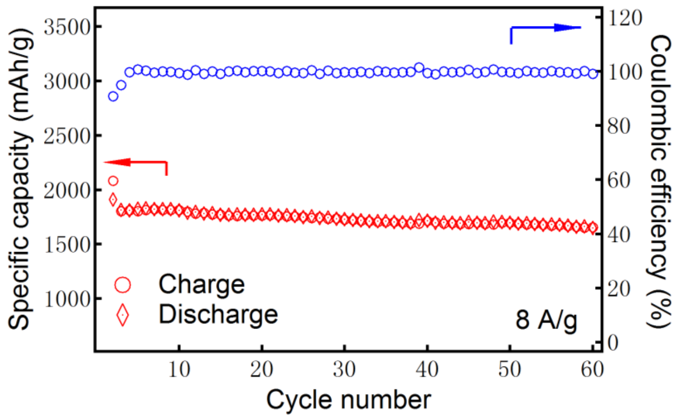

Figure S5. The results of cycling performance of the 1D@PSiNW anode tested at a current density of 8.0 A·g^{-1}.

Reference:

1. K. Peng, H. Fang, J. Hu, Y. Wu, J. Zhu, Y. Yan and S. Lee, *Chemistry-A European Journal*, 2006, **12**, 7942-7947.
2. K. Peng, J. Jie, W. Zhang and S.-T. Lee, *Appl. Phys. Lett.*, 2008, **93**, 033105.
3. W.-J. Yu, P.-X. Hou, L.-L. Zhang, F. Li, C. Liu and H.-M. Cheng, *Chem. Commun.*, 2010, **46**, 8576-8578.
4. P. Zhang, C. Shao, X. Li, M. Zhang, X. Zhang, Y. Sun and Y. Liu, *J. Hazard. Mater.*, 2012, **237-238**, 331-338.
5. G. Zheng, Y. Yang, J. J. Cha, S. S. Hong and Y. Cui, *Nano Lett.*, 2011, **11**, 4462-4467.
